# Bidirectional anti-tumor and immunological strategies by targeting GARP–TGF-β axis in adult T-cell leukemia/lymphoma

**DOI:** 10.1038/s41375-025-02725-0

**Published:** 2025-08-04

**Authors:** Kako Suzuki, Seina Kusayanagi, Yuta Kuze, Masato Hata, Shiho Kozuma, Koji Jimbo, Yasuhito Nannya, Yutaka Suzuki, Kaoru Uchimaru, Makoto Yamagishi

**Affiliations:** 1 Laboratory of Viral Oncology and Genomics, Department of Computational Biology and Medical Sciences, Graduate School of Frontier Sciences, The University of Tokyo, Tokyo, Japan; 2 Laboratory of Tumor Cell Biology, Department of Computational Biology and Medical Sciences, Graduate School of Frontier Sciences, The University of Tokyo, Tokyo, Japan; 3 Laboratory of Systems Genomics, Department of Computational Biology and Medical Sciences, Graduate School of Frontier Sciences, The University of Tokyo, Chiba, Japan; 4 Research Institute Munich, Daiichi Sankyo Europe GmbH, Munich, Germany; 5 Translational Research Department, Daiichi Sankyo Co., Ltd., Tokyo, Japan; 6 Department of Hematology and Oncology, The Institute of Medical Science, The University of Tokyo, Tokyo, Japan

**Keywords:** T-cell lymphoma, Drug development, Targeted therapies

## Abstract

In adult T-cell leukemia/lymphoma (ATL), tumor cells show a regulatory T-cell (Treg)-type phenotype, which influences their tumor immunity. However, our knowledge of what molecular events are involved in pathogenesis is still missing. Here, we took advantage of this unique phenotype and screened whole transcriptome data from primary ATL cells to search for effective therapeutic targets. Glycoprotein A repetitions predominant (GARP) was identified as a novel tumor antigen in ATL. ATL cells overexpress GARP and release transforming growth factor-β (TGF-β). The GARP–TGF-β axis promotes cell proliferation of ATL cells and human T-cell leukemia virus type 1 (HTLV-1)-infected cells with changes in cell signaling activities and shaping of Treg gene expression patterns, but suppresses the activity of surrounding effector T-cells. Remarkably, this study has provided a breakthrough therapeutic concept that achieves the dual effect of direct tumor cell depletion and indirect immune activation by a single treatment targeting GARP. DS-1055a, an anti-GARP monoclonal antibody, selectively and effectively depleted malignant ATL cells via antibody-dependent cellular cytotoxicity, supporting the proof-of-concept in the preclinical study. Our findings highlight the key to understanding the cell origin of ATL and developing unprecedented therapeutic strategies for refractory diseases.

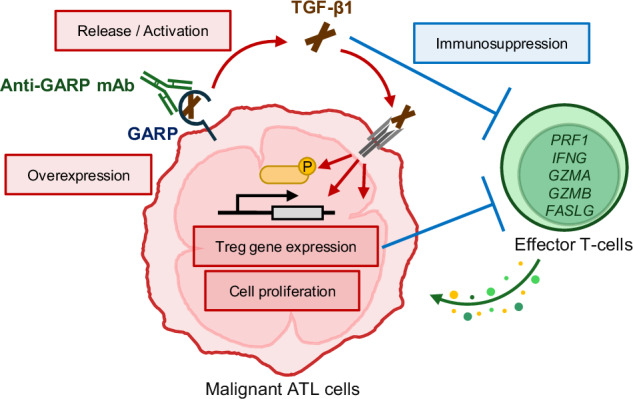

## Introduction

FOXP3^+^/CD25^+^/CD4^+^ regulatory T-cells (Tregs) and antitumor immune responses are inextricably linked, and transforming growth factor β (TGF-β), which is mainly secreted by Tregs, is an important immunosuppressor [1]. Tregs are generally abundant in the tumor microenvironment and play a critical role in the induction of tolerance to tumor antigens and the suppression of antitumor immunity. Therefore, tumor immunity can be enhanced by depleting Tregs [2]. Monoclonal antibodies (mAbs) or small molecules have targeted various molecules highly expressed by Tregs, including immune checkpoint molecules, cytokine and chemokine receptors, and metabolites. To date, however, Treg depletion therapies, such as targeting CD25 and CCR4, have not shown clear clinical benefits in clinical trials [3]. Furthermore, systemic Treg depletion should be considered because it increases the risk of immune-related adverse events [2, 4]. Therefore, therapeutic strategies that selectively deplete highly suppressive Tregs are necessary to adequately control antitumor immunity.

Adult T-cell leukemia/lymphoma (ATL) has an inferior prognosis and is a refractory malignancy caused by human T-cell leukemia virus type 1 (HTLV-1). Only approximately 5% of asymptomatic HTLV-1 carriers progress to ATL [5], with a median survival time of approximately 1 year [6], due to not only persistent HTLV-1 infection over several decades but also the associated accumulation of complex genetic, epigenetic, transcriptomic, and signaling abnormalities [7–10]. Treatment strategies for ATL vary by clinical subtype. Indolent ATL, including smoldering and favorable chronic types, progress relatively slowly and are usually managed with watchful waiting until disease progression. On the contrary, aggressive ATL, including unfavorable chronic, lymphoma, and acute types, progress rapidly and are often treated with conventional chemotherapy, a combination of interferon α and zidovudine, and allogeneic hematopoietic stem cell transplantation [11]. Recent advances in treatment modalities may produce a high response rate with minimal side effects in patients with ATL [12]. One approach is a mAb therapy, such as mogamulizumab [13] and brentuximab vedotin [14], which have advantages in specificity and durability, and the other approach is a mechanism-based therapy, exemplified by valemetostat [15], which targets the fundamental abnormalities of ATL identified through comprehensive data of primary ATL cells. However, relapse remains a significant problem, which underscores the urgent need for further research and development of well-developed treatment options.

ATL cells were the first neoplastic cells reported to express FOXP3, and two-thirds of ATL cases express FOXP3 [16, 17]. ATL cells also constitutively and highly express CD25 (IL-2Rα) [18]. HTLV-1 bZIP factor (HBZ), an antisense viral gene of HTLV-1, plays a role in the Treg phenotype of HTLV-1-infected and ATL cells through the induction of FOXP3 expression as a transcription factor, an increase in the Treg population, and enhancement of TGF-β signaling through interaction with Smad2/3 [19, 20]. Recent single-cell transcriptome analyses have suggested the involvement of the Treg phenotype and clonal proliferation of ATL cells in the activation or precancerous state, highlighting the functional heterogeneity and plasticity within the FOXP3^+^ Treg phenotype [21, 22]. Despite several reports indicating a possible association between the Treg phenotype and ATL, the origin of ATL cells remains unclear, and the underlying mechanisms for immunosuppression remain controversial [23, 24]. Notably, the most significant advantage of ATL is that the tumor cells themselves show a Treg-like phenotype, making it highly compatible with Treg-depleted-tumor immunotherapeutic strategies.

Considering the accumulated evidence and the remaining clinical challenges, we hypothesized that a mechanism-based therapeutic strategy simultaneously targeting both the intrinsic factors of ATL and those regulating immunity would be highly effective and durable. To identify novel therapeutic targets, the following criteria were set: (1) crucial for the cell lineage and function of ATL, (2) specific to ATL, and (3) tumor antigens suitable for antibody therapies in ATL.

In this study, we newly identified GARP as a tumor antigen, which fit the three criteria by screening clinical whole transcriptome data from HTLV-1 infection to disease onset. The GARP–TGF-β axis played a pivotal role in the proliferation and phenotyping of the tumor cells and immunosuppression in the ecosystem. By targeting the GARP–TGF-β axis, we presented a novel bidirectional strategy that can lead to tumor regression and immune activation.

## Methods

### Clinical samples, cell lines, and cell culture

Peripheral blood mononuclear cells (PBMCs) and plasma of HTLV-1 carriers and patients with ATL were provided from IMSUT Hospital, The Institute of Medical Science, the University of Tokyo, Japan. All patients were categorized into clinical subtypes according to Shimoyama’s criteria [25]. PBMCs were isolated by Lymphoprep separation (AXS). HTLV-1-uninfected PBMCs and CD4^+^ T-cells were obtained from Lonza. The HTLV-1-infected cell line MT-2 cells were provided by an established researcher, Dr. Miyoshi. HUT102 and C91/PL cells were provided by Dr. Fujisawa. ATL-derived cell TL-Om1 cells were provided by established researcher Dr. Sugamura. ATN-1 cells were purchased from the RIKEN BRC cell bank (RCB1440). HTLV-1-uninfected T-cell lines, Jurkat cells, were purchased from RIKEN BRC cell bank (RCB3052). HTLV-1-uninfected T-cell lines Jurkat (RCB3052) and CCRF-CEM (RCB1980) were purchased from the RIKEN BRC cell bank. MOLT-4 cells were purchased from ATCC (CRL-1582). HEK293FT cells were purchased from Thermo Fisher Scientific (R70007). These cell lines were verified by each cell bank or established researchers and monitored for cross-contamination. The cell lines were also tested for mycoplasma contamination using mycoplasma detection PCR (6601, Takara) and were negative for mycoplasma contamination. All lymphoma cell lines were cultured in RPMI1640 (GIBCO) with 10% fetal bovine serum (FBS, GIBCO) and antibiotics (GIBCO). HEK293FT cells were cultured in DMEM (Nissui, Japan) with 10% FBS and antibiotics. In the co-culture assay, activated PBMCs and engineered GARP^+^ ATN-1 cells were co-cultured in 0.4 µm pore size inserts (Thermo Scientific). All cell lines and primary cultures were maintained at 37 °C with 5% CO_2_.

### Antibodies and reagents

Recombinant human IL-2 (Peprotech) and/or IL-7 (Peprotech) was added to the co-culture assay at 10 ng/mL. Recombinant human TGF-β1 (Peprotech) was used at 10 ng/mL unless otherwise stated. The TGF-β type I receptor (ALK5) inhibitor SB431542 (Cayman Chemical) was used at 10 μM. Anti-TGF-β antibody (R&D) was used at 1 µg/mL. DS-1055a was produced in the Lonza/Biowa POTELLIGENT® CHOK1SV™ cell line to enhance the ADCC response and was provided by Daiichi Sankyo Co., Ltd., Tokyo, Japan, and used at 1 or 10 µg/mL.

### In vitro/ex vivo ADCC assay

Antibody-dependent cellular cytotoxicity (ADCC) assay was performed on an engineered GARP^+^ ATL cell line and PBMCs from patients with ATL and HTLV-1 carriers using DS-1055a in the presence or absence of effector PBMCs (in vitro) or NK cells (ex vivo) from healthy donors. The effector-to-target ratio was fixed at 50:1 (in vitro) or 2:1 (ex vivo). ADCC activities were evaluated by the detection of dead cells or the GARP^+^ cell population using flow cytometry.

### Flow cytometry

HTLV-1-infected cell populations and ATL cell populations were obtained using a HAS-flow method [26, 27]. For cell surface staining, PBMCs and engineered cell lines were stained with fluorescent-labeled antibodies; anti-CADM1-biotin (clone 3E1, MBL), anti-CD7-APC-eFluor780 (clone 124-1D1, Invitrogen), anti-CD4-PE/Cyanine7 (clone OKT4, BioLegend), anti-CD8-BB515 (clone RPA-T8, BD Biosciences), anti-CD56-Brilliant Violet 510 (clone HCD56, BioLegend), anti-GARP-APC (clone 7B11, BioLegend), anti-LAP-FITC (clone S20006A, BioLegend), anti-TIGIT-APC (clone A15153G, BioLegend), anti-PD-1-APC (clone EH12.2H7, BioLegend), and anti-TGF receptor II-Brilliant Violet 421 (clone W170551, BioLegend). After washing the cells, phycoerythrin (PE)-conjugated streptavidin (SA10041, Thermo Fisher Scientific) was applied. 7-AAD (BD Biosciences) was added to the same samples to stain dead cells 5 min before flow cytometry.

For intracellular staining, PBMCs were treated with BD Pharmigen Human FoxP3 Buffer Set according to the manufacturer’s directions and stained with fluorescent-labeled antibodies; anti-FOXP3-Brilliant Violet 421 (clone 206D, BioLegend), anti-Ki-67-APC (clone Ki-67, BioLegend), and anti-IFN-γ-Brilliant Violet 421 (clone 4S.B3, BD Biosciences) after initial surface staining. To detect intracellular cytokines, PBMCs were treated with a protein transport inhibitor (51-2092KZ, BD Biosciences). Fixable Viability Stain 575 V (565694, BD Biosciences) was added to the same samples before fixation to detect dead cells. Isotype control antibodies were used for appropriate gating.

The expression level of each molecule was evaluated by FACSymphony A1 (BD Biosciences). CD4^+^/CADM1^+^/GARP^+^ and CD4^+^/CADM1^+^/GARP^−^ cells were sorted by FACSAria III (BD Biosciences). The collected data were analyzed by FlowJo software (v10.10.0, Tree Star).

### Targeted capture sequencing

Genomic DNA from enriched ATL cell populations was extracted using the QIAamp DNA Blood Mini Kit (Qiagen). Target capture was conducted using the SureSelect Target Enrichment System (Agilent Technologies). To comprehensively cover genes involved in ATL, 280 human genes were selected, including 50 genes frequently mutated in ATL [7] and 190 genes frequently mutated in hematological and solid malignancies. The Agilent SureDesign web-based application was used for capture bait design as previously described [26]. The sequence data were obtained using the NovaSeq 6000 system (Illumina) with 100-bp paired-end reads. The sequenced data were aligned to the human reference genome hg38 by the BWA (v0.7.15) software. The PCR duplicates were removed using Picard (v2.92) and SAMtools (v1.2) software. Uninfected T-cells (CD4^+^/CADM1^−^/CD7^+^ “P” population) were used as matched normal controls to call somatic mutations. The somatic mutation candidates were called using MuTect2 from the GATK (v4.0.12) software and annotated with ANNOVAR (v20191024). Candidate mutations with (1) ≥5 variant reads in tumor samples, (2) a variant allele frequency (VAF) in tumor samples ≥0.01, (3) read depth ≥200, and (4) tumor variants with a normal variant ratio ≥2 were adopted and further filtered by excluding synonymous SNVs.

### Clonality analysis

The clonality analysis of HTLV-1-infected cells was performed by high-throughput sequencing-based mapping of proviral integration sites as described previously [26]. To designate the virus integration sites, sequence reads were aligned to the human reference genome hg38 and the virus genome (NC_001436.1) by BWA. Pair-end reads spanning the viral and human genomes and soft-clipped reads (>15 bp soft-clipped region) were extracted using Perl scripts and then validated by Blastn (v2.6.0+). Clonality was calculated as the population size of each clone by counting the extracted reads at host–provirus junction sites.

### Whole-exome sequencing

For somatic variant detection, whole-exome sequencing (WES) was carried out using next-generation sequencing by Azenta Japan Corporation. Twenty-five ng of genomic DNA from tumor and paired normal cells were enzymatically sheared into approximately 200 bp in size, followed by DNA library preparation using Twist Library Preparation EF Kit 2.0 (Twist Bioscience). Resulting libraries were quantified and qualified by Qubit dsDNA HS Assay (Thermo Fisher) and TapeStation D1000 ScreenTape (Agilent Technologies), and 4~8 individual libraries were then pooled in equal amounts. Pooled libraries were mixed and treated with a human exome probe set (Twist Exome 2.0 panel) to enrich exome regions using Twist Fast Hybridization and wash kit (Twist Bioscience). Post-enriched libraries were pooled/multiplexed at a specific ratio to generate 200× and 50× coverages for tumor and normal cells, respectively, and loaded onto a next-generation sequencing platform, NovaSeq 6000 (Illumina). Sequencing was carried out according to the manufacturer’s instructions with a 150 bp pair-end configuration. Image analysis and base calling were conducted by software on the NovaSeq instrument. Demultiplexing and FASTQ generation were performed by a bcl2fastq pipeline. Sequencing raw data was optimized by using Cutadapt software (version 1.9.1) to remove the primers and adapters. The Sentieon pipeline (https://www.sentieon.com/products/) was used to clean data and call somatic variations. Copy number variation was detected by Control-FREEC.

### RNA sequencing

RNA-seq data of enriched ATL cells from patients with ATL and HTLV-1-infected cells from carriers were obtained in a previous study [10]. Transcriptome data of other cell line models, in vitro infection models, Tregs, and GARP^+/−^ ATL cells were obtained in this study. Total RNA of each sample was extracted using TRIzol reagent (Invitrogen) and quantified and qualified by the Agilent 2100 Bioanalyzer (Agilent Technologies) and NanoDrop (Thermo Fisher Scientific). Of the total RNA with an RNA integrity number (RIN) value above 7, 20 ng was used following library preparation. The library preparation and sequencing were processed and analyzed by Genewiz. The libraries with different indices were multiplexed and loaded on an Illumina HiSeq instrument according to the manufacturer’s instructions (Illumina). Sequencing was carried out using a 2 × 150-bp paired-end configuration; image analysis and base calling were conducted by the HiSeq control software (HCS v2.2.38 or later) plus OLB plus GAPipeline-1.6 (Illumina) on the HiSeq instrument. For quality control, to remove technical sequences, including adapters, PCR primers or fragments thereof, and quality of bases lower than 20, the pass filter data of fastq format were processed by Trimmomatic (v0.30) to be high-quality clean data. For mapping, Hisat2 (v2.0.1) was used to index the reference genome sequence. Finally, clean data were aligned to the reference genome via the software Hisat2. Gene expression data were comparatively analyzed using transcripts per million (TPM). For differentially expressed gene analysis, HTSeq (v0.6.1) estimated gene and converted read counts to transcripts per million from the paired-end clean data. Selected genes were subjected to principal component analysis using the iDEP.91 pipeline that contains the DESeq2 package.

### ATAC sequencing

ATAC-seq data of enriched ATL cells from patients with ATL and HTLV-1-infected cells from carriers were obtained in a previous study [10].

### Single-cell RNA sequencing and ATAC sequencing

The scRNA-seq data of PBMCs from patients with ATL and HTLV-1 carriers were obtained in previous studies [15, 26]. The scATAC-seq data of PBMCs from patients with ATL were obtained in a previous study [15].

### Single-cell multiome analysis

The methods and data analysis pipeline of the single-cell multiome analysis were previously reported [15]. Briefly, the single-cell multiome (scMultiome) libraries were constructed by using Chromium Controller and 10x Genomics Chromium Next GEM Single Cell Multiome ATAC plus Gene Expression (CG000365 Rev C, CG000338 Rev F, 10x Genomics). The libraries were sequenced using the NovaSeq 6000 system (Illumina) according to the manufacturer’s instructions. For ATAC libraries, sequencing was performed using a 50 × 49-bp paired-end configuration. RNA library sequencing was performed using a 28 × 91-bp paired-end configuration. The scMultiome dataset was processed using Cell Ranger ARC v2.0.0 (Cell Ranger ARC, 10x Genomics). To remove batch effect, the scMultiome RNA dataset was processed by Seurat (v4.3.0) reciprocal principal component analysis. The scMultiome ATAC dataset was recounted by Signac (v1.9.0) using the merged peak bed files and processed by Harmony (v0.1.1). For the detection of virus reads, we processed the Cell Ranger GRCh38-aligned sequence data. The high-quality clean data were aligned to the human reference genome (hg38) and virus genome (NC_001436.1) via the software STAR. Chromium cellular barcode tags with virus reads were defined as having at least one virus read detected. Almost all virus-aligned reads were derived from the antisense strand. The extracted Chromium cellular barcode tags with virus reads were mapped on a UMAP projection using the Loupe Cell Browser.

### Phospho-protein array analysis

Cells were pre-cultured with RPMI1640 supplemented with 1% FBS overnight and then incubated for 2 h in the presence or absence of TGF-β1 (50 ng/mL). After stimulation, cells were resuspended in the extraction buffer supplemented with protease and phosphatase inhibitors and analyzed using the Phospho Explorer Antibody Array, which contains 1318 antibodies (#PEX100, Full Moon Biosystems), according to the protocol of the manufacturer. The data were normalized to the median signal intensity of all signals on the slide.

### Bioinformatic analysis and statistics

The Integrative Genomics Viewer tool was used for visualizing and interpreting the results of DNA-seq and RNA-seq. Gene set enrichment analysis was performed using GSEA software (v4.1.0) (http://www.broadinstitute.org/gsea) with 1000 permutations. Gene sets used in this study were selected from the MSigDB hallmark gene sets (http://www.broadinstitute.org/gsea/msigdb/collections.jsp). Significantly enriched gene sets were evaluated by normalized enrichment score (NES) and nominal *P*-value (*P* < 0.001). Gene ontology analysis was performed by DAVID Bioinformatics Resources (https://david.ncifcrf.gov/). The nucleotide sequence of the human *LRRC32* promoter region was inspected with JASPAR transcription factor binding sites searching software (https://jaspar.elixir.no/) for the presence of putative binding sites of FOXP3, NFAT, and NF-κB with a relative score profile threshold of 80%. Significant differences in gene expression and other biological assays between the two groups were analyzed by a two-sided Student’s *t*-test. Adjustments were not made for multiple comparisons. Correlations between two groups were analyzed by a two-sided *Pearson*’s correlation coefficient, and probabilities of overlap between gene sets were statistically tested.

### Data visualization

Box plots, beeswarm plots, and hierarchical clustering were analyzed and visualized by using R (v4.2.3) and GraphPad PRISM 10 (Dotmatics). Box plots are defined as follows: the middle line corresponds to the median; the lower and upper hinges correspond to the first and third quartiles. The upper whisker extends from the hinge to the largest value no further than 1.5 × IQR from the hinge (where IQR is the inter-quartile range or distance between the first and third quartiles). The lower whisker extends from the hinge to the smallest value at most 1.5 × IQR of the hinge. All data points are overlaid on the box plot.

### HTLV-1 infection through a co-culture system

In vitro HTLV-1 infection was established by co-culture of normal CD4^+^ T-cells and mitomycin C (Nacalai Tesque) pre-treated MT-2 or HUT102 cells at a 2:1 ratio. Cell population size of the HTLV-1-infected cells was evaluated by measurement of HTLV-1 proviral load, as described previously [28]. Briefly, quantitative multiplex real-time PCR (qRT-PCR) was performed with two sets of primers specific for the HTLV-1 provirus and the human gene encoding the RNase P enzyme. The proviral loads were expressed as copy numbers per 100 PBMCs, based on the assumption that infected cells harbored one copy of the integrated HTLV-1 provirus per cell.

### Lentiviral vector construction and transfection of recombinant lentivirus

For knockdown of *LRRC32* (GARP), a replication-defective, self-inactivating lentivirus vector (CS-H1-Venus-IRES-Bsd) was used (Riken, BRC). We designed three shRNA sequences (Supplementary Table 1) and cloned them into CS-RfA-EVBsd via pENTR4-H1. For stable expression of GARP, an *LRRC32*-encoded lentivirus vector was purchased from VectorBuilder (pLV-EGFP-Puro-EF1A-hLRRC32). The established viral vectors were co-transfected with the packaging plasmid (pCAG-HIVgp) and the VSV-G- and Rev-expressing plasmid (pCMV-VSV-G-RSV-Rev) into HEK293FT cells. High-titer viral solutions were prepared by centrifugation-based concentration and used for transduction into cell lines. The infection was attained by the spinoculation method and then cultured under appropriate conditions for 5–7 days. Blasticidin (10 µg/mL) or Puromycin (0.5 µg/mL) was used to select the transduced population. Expression levels of fluorescent proteins (Venus and EGFP) and GARP were evaluated by flow cytometry using BD FACSymphony A1. Alternatively, knockdown efficiencies were evaluated by qRT-PCR with a specific primer set (Supplementary Table 1).

### Measurement of TGF-β1

Cell supernatants were collected following 72 h (cell lines) or one week (PBMCs) incubation. After converting latent TGF-β1 to active TGF-β1 by acidification, the concentrations of secreted TGF-β1 in the cell supernatants were measured using Cytometric Bead Array (CBA) Human TGF-β1 Single Plex Flex Set according to the manufacturer’s directions (BD Biosciences). CBA samples were evaluated by BD FACSymphony A1. Total TGF-β1 in plasma was measured after activation using a human Quantikine ELISA kit according to the manufacturer’s directions (R&D Systems).

### Cell proliferation assay

For evaluation of the proliferative effects of TGF-β, cell lines (1 × 10^4^–10^5^) were plated in a 48-well plate with RPMI1640 supplemented with 1% FBS in the presence or absence of TGF-β1 (10 ng/mL). Primary ATL cells (4 × 10^5^) were plated in a 24-well plate with RPMI1640 supplemented with 20% FBS and IL-2 (10 ng/mL) in the presence or absence of TGF-β1 (10 ng/mL). After incubation for one week, the number of cells was counted using a hemocytometer. For evaluation of the proliferative effects by GARP knockdown and ectopic expression, transduced C91/PL cells (1 × 10^5^) were plated in a 12-well plate with RPMI1640 supplemented with 10% FBS for 3 days. The cell numbers were evaluated by Cell Counting Kit-8 (WST-8 assay, Dojindo) following the manufacturer’s protocol.

### Western blotting

Whole cell lysates were prepared with RIPA buffer (10 mM Tris-HCl, pH 7.4, 1% Triton X-100, 0.1% sodium deoxycholate, 0.1% SDS, 150 mM NaCl, 1 mM EDTA, Protease inhibitor cocktail, Phosphatase inhibitor cocktail). Protein concentration was measured by Bio-Rad Protein Assay. The lysates (20 µg protein) were separated by SDS-polyacrylamide gel electrophoresis and transferred to a polyvinylidene difluoride membrane. After blocking with 5% non-fat dry milk, the membrane was immunoblotted with the indicated primary antibodies; anti-Smad2/3 (clone D7G7, Cell Signaling Technology) and anti-phospho-Smad2 (S465/467) / Smad3 (S523/425) (clone D27F4, Cell Signaling Technology), followed by HRP-linked donkey anti-rabbit IgG (Cytiva). The membrane was finally visualized by enhanced chemiluminescence (ECL) Prime Western Blotting Detection Reagents (Cytiva) and analyzed using ChemiDoc XRS Plus (Bio-Rad).

## Results

### Distinctive Treg-type gene expression profiles in ATL and asymptomatic HTLV-1 carriers

**Fig. 1:**
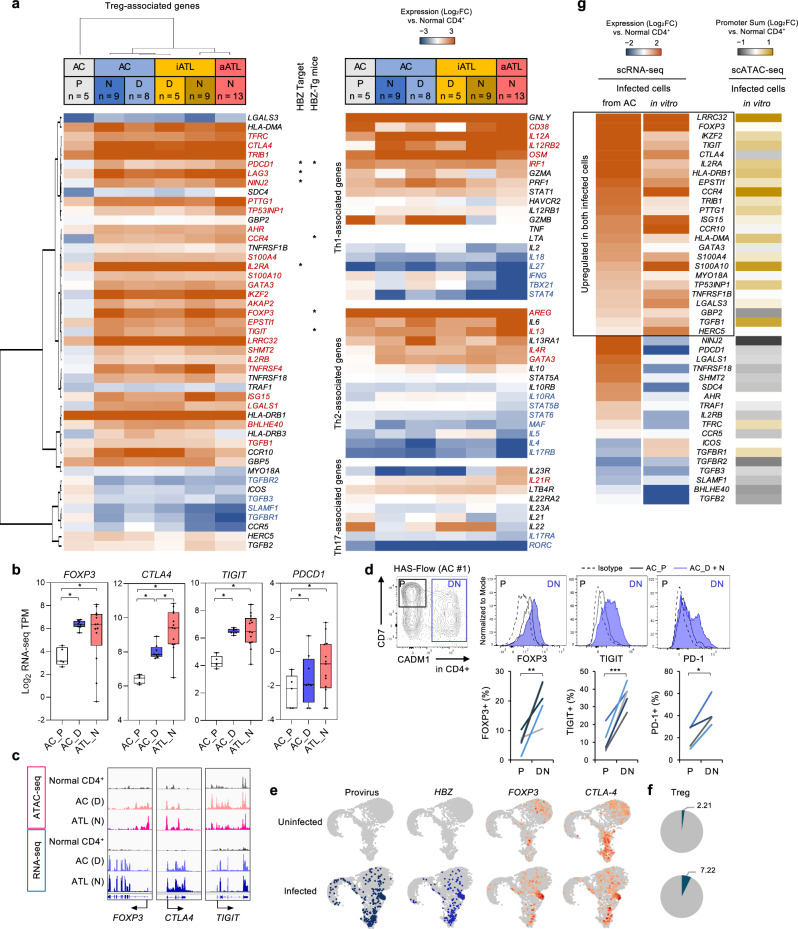
Abnormal Treg-type gene expression profiles in ATL and pre-onset stage. **a** Heatmaps and hierarchical clustering of expression patterns of genes associated with Treg, Th1, Th2, and Th17 cells in uninfected T-cells (“P”, CD4^+^/CADM1^−^/CD7^+^), HTLV-1-infected T-cells (“D”, CD4^+^/CADM1^+^/CD7^+^; “N”, CD4^+^/CADM1^+^/CD7^−^) from asymptomatic carriers (AC, *n* = 13), patients with indolent type ATL (iATL, *n* = 9) and acute type ATL (aATL, *n* = 13). Gene symbols with significant differences between aATL_N and normal CD4^+^ T-cells are shown in red (upregulated) or blue (downregulated) (*P* < 0.05). “HBZ Target” is defined by HBZ-ChIP-seq peaks within ±10 Kb from the transcription start site [29]. “HBZ-Tg mice” represents the top 50 genes upregulated in HBZ transgenic mice [30]. **b** Box plots show gene expression levels of representative Treg genes. **c** IGV tracks depict peaks of ATAC-seq and RNA-seq data at representative Treg loci. **d** FOXP3, TIGIT, and PD-1 staining of PBMC from AC #1 gated on “P” and “D” + “N” subpopulations. Line graphs show FOXP3, TIGIT, and PD-1 positive cells (%) in PBMC from five ACs. **e**–**g** Single-cell analysis on an in vitro infection model. UMAP projection shows single-cell multiome ATAC plus gene expression data in uninfected and infected groups, with cells colored according to provirus, *HBZ*, *FOXP3*, and *CTLA4* expression (**e**). The pie chart shows the percentage of Treg cell population (**f**). Heatmaps show expression pattern (left) and promoter activity (right) of Treg-associated genes in infected cells from carriers or de novo infected cells in vitro (**g**). *P < 0.05, **P < 0.01, ***P < 0.001 (two-sided Student’s *t*-test).

We previously obtained whole transcriptome data from ATL cells and premalignant HTLV-1-infected cells [10]. Detailed analysis of genes associated with the T-cell lineage from infection to pathogenesis sheds light on the origin and lineage of ATL cells. Using expression data from asymptomatic carriers (AC, *n* = 13) and patients with indolent type ATL (iATL, *n* = 9) or acute type ATL (aATL, *n* = 13), hierarchical clustering based on T-cell subset-associated genes revealed distinctive Treg-type gene expression profiles in ATL and HTLV-1-infected cells in carriers (Fig. 1a; Supplementary Fig. 1a). Among the Treg gene set, 57% of the genes showed significantly increased expression in ATL cells (*P* < 0.05). Some of these genes have been recognized as targets of the HTLV-1 protein HBZ [29, 30]. Gene sets in other T-cell subsets (Th1, Th2, and Th17) did not show significant differences. Principal component analysis (PCA) using the Treg gene set also revealed that ATL and infected cells differed from uninfected T-cells (Supplementary Fig. 1b). Comparative analysis of each case showed Treg gene enrichment in all cases (Supplementary Fig. 1c). ATL cases formed a distinct cluster separately from carriers. Some carrier cases with high PVL (8.29%–25.18%) were also part of the ATL cluster, suggesting that Treg gene expression profiles are associated with the proliferation of infected cells.

ATL and premalignant infected cells showed significant upregulation of the Treg hallmark genes essential for Treg stability and function, including *FOXP3*, *CTLA4*, *TIGIT*, and *PDCD1* (Fig. 1b). Using ATAC-seq data [10], several open chromatin regions, such as *FOXP3*, *CTLA4*, and *TIGIT*, were detected, indicating that the increased expression of Treg genes was associated with changes in the abnormal chromatin structure (Fig. 1c). Flow cytometry analysis detected the expression of FOXP3, TIGIT, and PD-1 proteins in some of the infected cells (Fig. 1d). The expression of Treg genes was higher in ATL cells than in premalignant infected cells (Fig. 1b; Supplementary Fig. 1c, d). These results indicated that the Treg gene expression pattern has already been established from premalignant states and that this is a crucial characteristic underlying ATL cells.

To investigate the mechanism of the Treg-type gene expression, we also performed single-cell analysis (GEX-ATAC) on an in vitro infection model. Co-culture of CD4^+^ T-cells and HTLV-1-infected cell line showed the upregulation of *FOXP3* and *CTLA4* in the infected cell cluster, resulting in increased Treg population (Fig. 1e, f). Several Treg genes were significantly upregulated in the de novo infected cell cluster as high as in infected cells from carriers (Fig. 1g). ATAC data were further obtained from the same experiment, and Treg gene expression showed correlation with the open chromatin structure (Fig. 1g). Thus, the Treg gene expression pattern was established in the early stages of the latency period, and this might be partially caused by HBZ and changes in the abnormal chromatin structure. These results suggested that Treg-type gene expression profiles are essential for the characteristics of ATL cells.

### GARP (*LRRC32*) is overexpressed in HTLV-1-infected and ATL cells

**Fig. 2:**
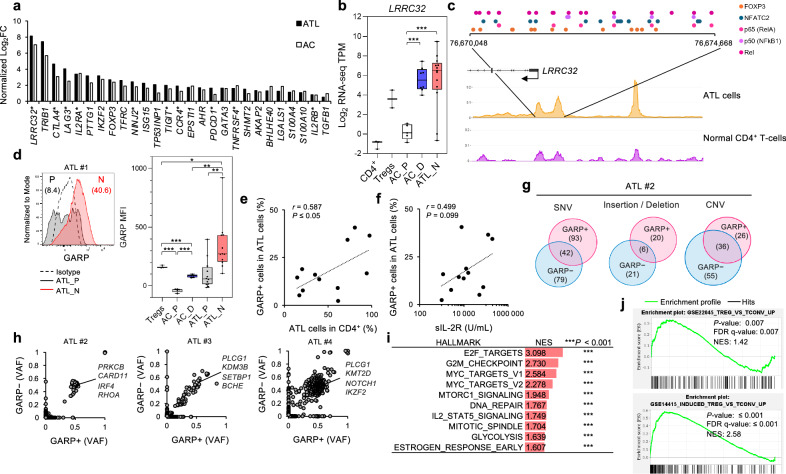
Identification of GARP as a tumor antigen. **a** Significantly upregulated Treg genes in ATL cells compared to normal CD4^+^ T-cells. Genes encoding proteins localized on the cell surface are marked with *. **b** Box plot shows expression level of *LRRC32* in normal CD4^+^ T-cells (*n* = 3), Tregs (*n* = 2), uninfected T-cells (AC_P, *n* = 5), infected cells from carriers (AC_D, *n* = 8), and ATL cells (ATL_N, *n* = 13). **c** ATAC peaks at the *LRRC32* locus in the ATL cell population or the uninfected CD4^+^ T-cell population from a patient with ATL. Upper colored dots represent predicted binding sites for each transcription factor. **d** Histogram shows GARP expression and positivity (%) in PBMC from ATL#1 gated on “P” and “N” subpopulations. Box plot shows GARP MFI in Tregs (CD4^+^/CD25^+^/CD127^low^, *n* = 3) from healthy donors, AC_P (*n* = 5), AC_D (*n* = 5), ATL_P (*n* = 12), and ATL_N (*n* = 12). **e** Scatter plot shows the correlation between ATL cells in CD4^+^ T-cells (%) (x-axis) and GARP^+^ cells in ATL cells (%) (y-axis). **f** Scatter plot shows the correlation between soluble IL-2R level (x-axis) and GARP^+^ cells in ATL cells (%) (y-axis). Correlation coefficients (*r*) are provided in the graphs. **g**, **h** Result of WES. Venn diagrams show somatic variants [single-nucleotide variant (SNV), insertion/deletion, copy number variation (CNV)] in representative GARP^+^ or GARP^−^ ATL cells (ATL #2, **g**). Scatter plots show variant allele frequencies (VAF) of somatic SNVs in GARP^+^ and GARP^−^ cells in three ATL cases (**h**). **i**, **j** Hallmark gene set enrichment analysis (GSEA) based on RNA-seq data in GARP^+^ ATL cells compared to GARP^−^ ATL cells in three ATL cases. For all pathways shown, significantly enriched gene sets were evaluated by normalized enrichment score (NES) and nominal *P*-value (**i**, ***P < 0.001, *n* = 3). Gene signatures related to Treg, significantly enriched in GARP^+^ ATL cells, are shown (**j**). *P < 0.05, **P < 0.01, ***P < 0.001 (two-sided Student’s *t*-test).

New factors that are tumor surface antigens and critical for the characteristic and function of the Treg-like phenotype of ATL were searched as targets for novel antibody therapies. We identified *LRRC32*, which encodes glycoprotein A repetitions predominant (GARP), as the top-most overexpressed Treg gene in ATL cells, consistent with a previous study in a small sample cohort exhibiting high GARP expression [26] (Fig. 2a, b). *LRRC32* was also overexpressed in premalignant infected cells. The chromatin structures at the *LRRC32* locus were examined using single-cell ATAC-seq data, and two abnormal open chromatin regions were detected at the *LRRC32* promoter region (Fig. 2c; Supplementary Fig. 2a). Several binding sites for the Treg transcription factors FOXP3, NFAT, and NF-κB were predicted in the open chromatin regions, consistent with previous reports [31]. Flow cytometry analysis of clinical specimens demonstrated that the GARP antigen, a marker of activated Tregs, was ectopically expressed in ATL and preleukemic cells (Fig. 2d). GARP surface protein was overexpressed on tumor cells in typical patients with aggressive ATL compared to Tregs (Fig. 2d). The GARP expression on the cell surface was positively correlated with the tumor population size (CD4^+^/CADM1^+^/CD7^−^; “N” fraction) and soluble IL-2R, suggesting that ATL cases with high GARP^+^ ATL cells may be clinically more aggressive (Fig. 2e, f). To verify this hypothesis, targeted deep sequencing [26] was performed for tumor cells from three cases with particularly high expression of surface GARP. All three cases showed a monoclonal expansion and multiple somatic mutations with high variant allele frequencies (Supplementary Fig. 2b).

To explore the characteristics of GARP^+^ ATL cells, GARP^+^ and GARP^−^ cell populations were separated from the peripheral blood mononuclear cells (PBMCs) of patients with ATL (*n* = 3) by flow cytometry. Whole-exome sequencing (WES) revealed that GARP^+^ and GARP^−^ ATL cells shared major genetic abnormalities frequently acquired in ATL [7] (Fig. 2g, h). On the contrary, the genetic abnormalities characteristic of each population were also observed, proposing that the subclonal structures were organized within the tumor. Furthermore, RNA-seq detected differentially expressed genes in GARP^+^ ATL cells compared with negative cells. Gene set enrichment analysis showed significant enrichment of genes associated with the cell cycle in GARP^+^ ATL cells. The high activation state of GARP^+^ ATL cells was supported by the enriched E2F and Myc target genes (Fig. 2i). GARP^+^ ATL cells were also significantly enriched for the immunosuppressive genes that are specifically upregulated in Tregs, suggesting that GARP plays a critical role in the induction of Tregs (Fig. 2j). These results indicate that GARP is an important antigen in the proliferation and phenotype of ATL cells.

### GARP promotes the release of mature TGF-β1 and cell proliferation

**Fig. 3:**
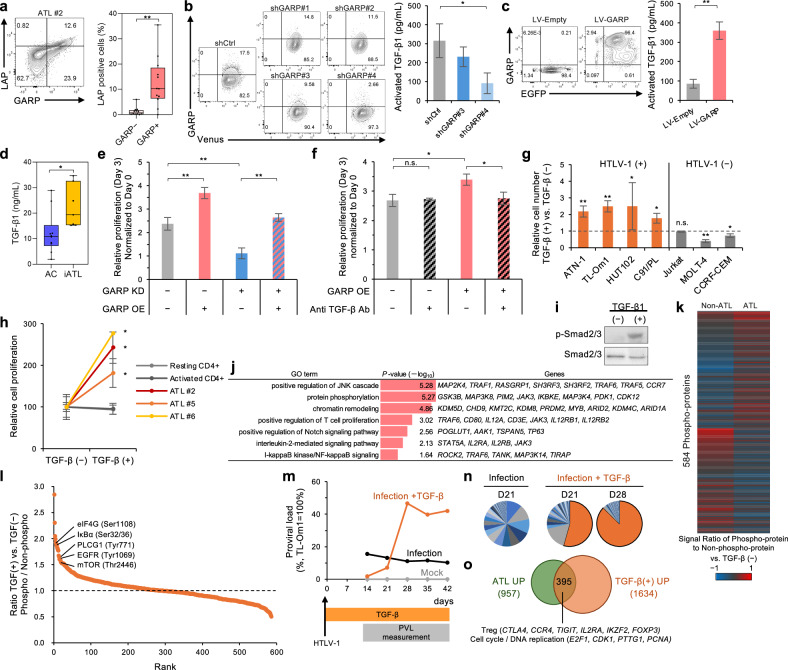
GARP–TGF-β pathway promotes the proliferation of both HTLV-1-infected and ATL cells. **a** GARP and LAP staining of PBMC from ATL #2 gated on “N” subpopulation. Box plot shows LAP^+^ cells (%) in GARP^−^ and GARP^+^ cells from patients with ATL (*n* = 12). **b** Knockdown of GARP in C91/PL cells. Cell surface GARP expression was evaluated by flow cytometry. The bar graph shows the amount of secreted, activated TGF-β1 (pg/mL) in culture supernatant measured by CBA (*n* = 3, mean ± SD). **c** Overexpression of GARP in ATN-1 cells. The bar graph shows the amount of activated TGF-β1 (pg/mL) (*n* = 3, mean ± SD). **d** The Box plot shows plasma TGF-β1 (ng/mL) in AC (*n* = 10) and iATL (*n* = 7). **e** A rescue experiment in C91/PL cells using simultaneous knockdown of GARP with shGARP#4, which targets the 3′ UTR (GARP KD), and ectopic expression of the GARP coding sequence via lentiviral vector (GARP OE). The bar graph shows relative cell proliferation at day 3, normalized to day 0 (*n* = 3, mean ± SD). **f** A neutralization experiment in C91/PL cells with ectopic expression of GARP (GARP OE) using an anti-TGF-β1/2/3 antibody (Anti TGF-β Ab). The bar graph shows relative cell proliferation at day 3, normalized to day 0 (*n* = 3, mean ± SD). **g** The effect of TGF-β on the cell proliferation of HTLV-1 positive cell lines (ATN-1, TL-Om1, HUT102, C91/PL) and HTLV-1 negative cell lines (Jurkat, MOLT-4, CCRF-CEM) cultured under 1% FBS condition (*n* = 3, mean ± SD). **h** The effect of TGF-β on the cell proliferation of primary ATL cells (*n* = 3), resting CD4^+^ T-cells, and activated CD4^+^ T-cells cultured with 20% FBS and 10 ng/mL IL-2 (*n* = 3, mean ± SD). **i**, **j** Effects of TGF-β. Western blots show total and phosphorylated Smad2/3 levels with or without TGF-β1 in TL-Om1 cells (**i**). The bar graph shows the results of gene ontology analysis of 1317 genes upregulated by TGF-β1 in TL-Om1 cells (**j**, TPM > 5, Log_2_FC > 1). **k**, **l** Comprehensive phosphorylation array. Heatmap shows the signal ratio of phospho-protein to non-phospho-protein in non-ATL cells (Jurkat, MOLT-4) and ATL cells (TL-Om1, ATN-1) after 2 h of TGF-β stimulation (**k**). Rank plot shows phosphorylated proteins by TGF-β in TL-Om1 cells (**l**). **m**–**o** In vitro infection model. The HTLV-1 co-culture assay was performed in the presence or absence of TGF-β. The kinetics of the de novo infected cell population was assessed by provirus quantification based on TaqMan real-time PCR (**m**). Pie charts represent clonalities of infected cells (**n**). A Venn diagram shows the overlap between upregulated genes in the de novo infected cell population with TGF-β at day 28 (orange circle, TPM > 5, Log_2_FC > 1 vs. Mock) and overexpressed genes in aATL (green circle, TPM > 5, Log_2_FC > 1 vs. normal CD4^+^ T-cells, *P* < 0.05) (**o**). *P < 0.05, **P < 0.01 (two-sided Student’s *t*-test).

GARP tethers latent TGF-β (latency-associated peptide, LAP) on the surface of Tregs and plays a crucial role in the production and release of active TGF-β. In this study, the cell surface expression of GARP/LAP was analyzed by flow cytometry, which revealed a significant increase of LAP in GARP^+^ ATL cells (Fig. 3a). To further analyze the production of active TGF-β by GARP expression in ATL cells, an engineered GARP^−^ cell line was established using specific shRNAs, and active TGF-β1 was measured in the culture supernatant. GARP depletion significantly downregulated the secretion of active TGF-β1 (Fig. 3b; Supplementary Fig. 3a). Moreover, a GARP-high ATL cell line was established, and a significant increase in active TGF-β1 secretion was detected (Fig. 3c). These results showed that GARP plays a role in the release of active TGF-β1 in ATL cells. We further measured TGF-β1 levels in clinical specimens and found that the amount of TGF-β1 in plasma from patients with ATL was higher than that in the plasma from carriers, suggesting that plasma TGF-β levels may increase as the disease progresses (Fig. 3d).

GARP depletion also blocked cell proliferation (Fig. 3e; Supplementary Fig. 3b). Ectopic expression of GARP in knockdown cells fully restored proliferation to control levels, supporting the functional importance of GARP in cell growth (Fig. 3e; Supplementary Fig. 3c). To elucidate the involvement of TGF-β in GARP-mediated proliferation, we performed a neutralization experiment using an anti-TGF-β1/2/3 antibody. Ectopic expression of GARP significantly enhanced cell proliferation, and this effect was canceled by TGF-β neutralization (Fig. 3f; Supplementary Fig. 3d). Collectively, these results indicate that GARP promotes cell proliferation through TGF-β release in HTLV-1-infected and ATL cells.

### TGF-β1 induces Treg-like phenotype and promotes proliferation of both HTLV-1-infected and ATL cells

Then, TGF-β was activated by GARP, and its function was examined in HTLV-1-infected and ATL cells. Proliferation assays revealed that TGF-β significantly promoted the proliferation of HTLV-1-infected and ATL cells under serum-starved conditions, whereas it hardly affected or blocked the proliferation of non-ATL T-cells (Fig. 3g). TGF-β also promoted the proliferation of primary ATL cells (Fig. 3h). The clonal structure of the cell population was not changed after TGF-β-dependent cell proliferation, suggesting that both ATL cells and their background infected cells commonly proliferated in response to TGF-β (Supplementary Fig. 3e). Therefore, the effects of TGF-β on signaling activation and transcriptome in ATL cells were investigated. Flow cytometric analysis confirmed that ATL cells expressed TGF-β receptor II (TGF-βRII) (Supplementary Fig. 3f). TGF-β1 treatment activated the canonical TGF-β signaling pathway represented by the phosphorylation of Smad2/3 and induced the expression of genes involved in T-cell proliferation, such as *IL2RA*, *IL2RB*, and *JAK3* (Fig. 3i, j; Supplementary Fig. 3g). Gene ontology analysis also identified multiple pathways in addition to the Smad signaling pathway in the TGF-β treated ATL cells, suggesting that the activation of noncanonical pathways may cause differences in the cell proliferation response between non-ATL and ATL cells. Therefore, a comprehensive phosphorylation array was performed, which revealed differences in the overall phosphorylation pattern of TGF-β (Fig. 3k). Multiple phosphorylation events of proteins involved in cell proliferation-related signaling pathways, including mTOR, EGFR, and IκBα (TCR/NF-κB pathways), were noted, which are strongly activated in ATL cells (Fig. 3l).

To further investigate TGF-β function in the early stage of leukemogenesis, an in vitro infection model was used. Polyclonal infected cell populations emerge during co-culture with CD4^+^ T-cells and HTLV-1-infected cells. One de novo infected clone was significantly expanded and proliferated by TGF-β (Fig. 3m, n). RNA-seq analysis showed that TGF-β induced the expression of genes highly expressed in ATL, including Treg genes such as *CTLA4*, *TIGIT*, and *FOXP3* (Fig. 3o). Collectively, these results indicate the involvement of the GARP–TGF-β axis in the induction of cell proliferation and shaping of Treg gene expression patterns.

### GARP expression in ATL cells suppresses the activity of the surrounding effector T-cells via TGF-β secretion

**Fig. 4:**
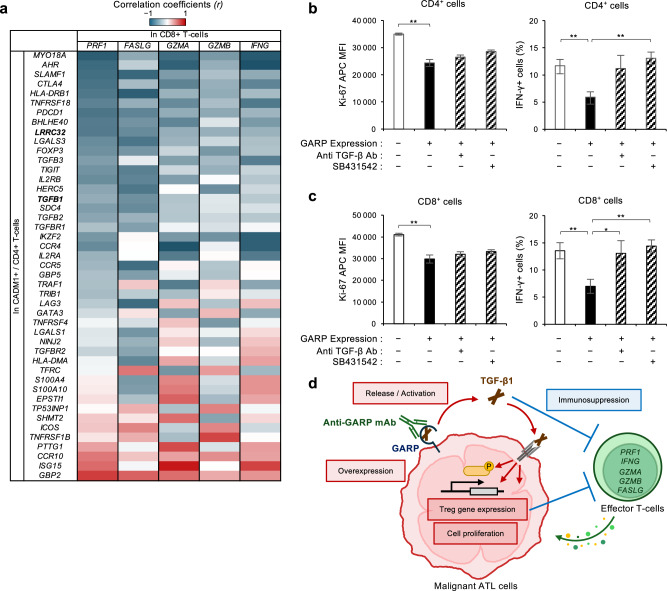
GARP suppresses the activity of effector T-cells mediated by TGF-β. **a** Heatmap depicts correlation coefficients (*r*) between expression levels of Treg genes in CD4^+^/CADM1^+^ ATL cell clusters and effector genes (*PRF1, FASLG, GZMA, GZMB*, and *IFNG*) in CD8^+^ T-cell clusters from single-cell RNA-seq data in patients with ATL (*n* = 3) and carriers (*n* = 3). **b**, **c** Co-culture of activated PBMCs and engineered GARP^+^ ATN-1 cells in the absence or presence of SB431542 or anti-TGF-β1 Ab. Ki-67 and IFN-γ staining gated on CD4^+^ and CD8^+^ subpopulations. Bar graphs show Ki-67 MFI (left) and IFN-γ positive cells (%) in CD4^+^ cells (**b**) and CD8^+^ cells (**c**). *n* = 3, mean ± SD. *P < 0.05, **P < 0.01 (two-sided Student’s *t*-test). **d** Schematic diagram showing the role of GARP–TGF-β axis in cell proliferation and immunosuppression in ATL.

TGF-β is a multipotent immunosuppressive cytokine [1]. In this study, the effect of GARP–TGF-β on effector T-cells in antitumor immunity was investigated by evaluating the T-cell activation level. Single-cell RNA-seq data from patients with ATL and HTLV-1 carriers [15, 26] were reanalyzed to determine whether CD8^+^ T-cell-mediated cytotoxicity was suppressed in vivo. The mRNA expression levels of key effector cytokines such as perforin, granzyme A, granzyme B, IFN-γ, and the Fas ligand in CD8^+^ T-cells were overall negatively correlated with that of Treg genes in CADM1^+^-tumor cells and infected cells, supporting the importance of the Treg-type transcriptome in the immune regulation of ATL (Fig. 4a). Indeed, with TGF-β as an immunoregulator, the expression levels of Ki-67 and IFN-γ decreased in a TGF-β1 concentration-dependent manner in normal PBMCs, indicating that TGF-β attenuates the activities in both CD4^+^ and CD8^+^ T-cells (Supplementary Fig. 4a). To further investigate the effect of TGF-β secreted from the cell surface GARP, activated PBMCs were co-cultured with GARP^+^ ATN-1 cell line in Transwell® with pore size, which did not allow cell–cell contact. The results showed that the expression levels of Ki-67 and IFN-γ were significantly decreased in both CD4^+^ and CD8^+^ T-cells in the presence of GARP^+^ cells (Fig. 4b, c). The decreased expression of IFN-γ in CD8^+^ T-cells was significantly restored by anti-TGF-β1/2/3 antibody or the TGF-β signaling inhibitor SB431542. These treatments also tended to restore the expression of Ki-67 in CD4^+^ and CD8^+^ T-cells. Thus, the results support that GARP expression in ATL cells is associated with immunosuppression via TGF-β secretion.

### Depletion of GARP^+^ ATL cells by DS-1055a, an anti-human GARP mAb

**Fig. 5:**
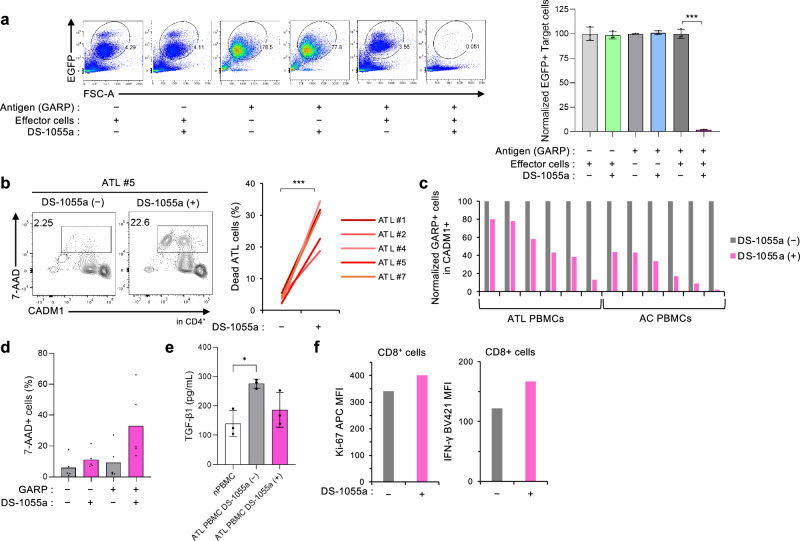
DS-1055a induces robust ADCC and depletes aggressive ATL cells. **a** ADCC assays were performed on an EGFP-labeled GARP^−^ and GARP^+^ ATN-1 cells using DS-1055a in the presence of effector PBMCs from healthy donors. ADCC activities were evaluated by detection of the EGFP^+^ cell population using flow cytometry (*n* = 3, mean ± SD). **b** ADCC assays were performed on PBMCs from patients with ATL (*n* = 5) using DS-1055a in the presence of effector NK cells from healthy donors. ADCC activities were evaluated by the detection of the dead ATL cells (CD4^+^/CADM1^+^/7-AAD^+^). **c** ADCC assays were performed on PBMCs from patients with ATL (*n* = 6) and carriers (*n* = 6) using DS-1055a. Autologous ADCC activities were evaluated by the detection of GARP-positive cells (%) within CD4^+^/CADM1^+^ cells. **d** The bar graph shows ADCC effects on GARP^+^ and GARP^−^ ATL cells in the allogeneic ADCC assays. **e** ADCC assays were performed on PBMCs from patients with ATL (*n* = 3) using DS-1055a. The bar graph shows the amount of secreted, activated TGF-β1 (pg/mL) in culture supernatant measured by CBA. Supernatants of normal PBMCs from healthy donors (*n* = 3) cultured for the same time were used as controls. (*n* = 3, mean ± SD). **f** ADCC assays were performed on fresh PBMCs from a patient with ATL (*n* = 1) using DS-1055a. Ki-67 and IFN-γ staining gated on CD8^+^ subpopulations. Bar graphs show Ki-67 and IFN-γ MFI in CD8^+^ cells. Due to the limited availability of fresh PBMCs, the sample size was small, and statistical analysis was not performed. *P < 0.05, ***P < 0.001 (two-sided Student’s *t*-test).

GARP is a surface antigen specifically expressed on tumor cells and Tregs, and the importance of the GARP–TGF-β axis in cell proliferation and immunosuppression in ATL was highlighted (Fig. 4d). Molecular targeted therapy against the GARP-based mechanism is expected to show the dual effect of directly depleting ATL cells and activating antitumor immunity. To address this issue, the efficacy of an anti-human GARP mAb (DS-1055a) for targeting ATL cells was tested. In vitro antibody-dependent cellular cytotoxicity (ADCC) assays revealed that DS-1055a induced robust and specific ADCC and depleted a GARP^+^ ATL cell line in the presence of effector cells (Fig. 5a). In addition, ex vivo allogeneic ADCC assays targeting tumor cells from aggressive ATL cases (*n* = 5) showed significant cell death by DS-1055a in all cases in a time-dependent manner (*P* < 0.001; Fig. 5b; Supplementary Fig. 5a, b). DS-1055a was effective against highly malignant tumor clones harboring multiple somatic mutations (Supplementary Fig. 2b). Furthermore, the autologous ADCC assay showed that DS-1055a depleted GARP^+^/CADM1^+^ ATL cells (*n* = 6) and preleukemic cells from carriers (*n* = 6) in all cases (Fig. 5c). Although DS-1055a induced significant cell death in GARP^+^ ATL cells as expected, it also induced cell death in some of GARP^−^ ATL cells (Fig. 5d; Supplementary Fig. 5c). This could be due to the plasticity of the surface GARP expression (Supplementary Fig. 5d). Since GARP^+^ ATL cells are the principal source of the immunosuppressive cytokine TGF-β, we evaluated the effects of DS-1055a on TGF-β level and surrounding immune cells. DS-1055a inhibited TGF-β release in ATL PBMCs, which secrete more TGF-β than normal PBMCs (Fig. 5e). Furthermore, ex vivo autologous ADCC assay showed that DS-1055a also enhanced CTL activity (Fig. 5f). Collectively, DS-1055a has the potential to deplete malignant ATL cells, regardless of the transient GARP expression level.

## Discussion

Targeting Tregs in cancer therapy may lead to new strategies to correct inappropriately restrained cancer immunity. ATL cells are inseparable from Tregs owing to their phenotype and functions. However, clinical applications utilizing the ATL-specific Treg phenotype have yet to be successful. In this study, we performed a comprehensive screen of transcriptome data and identified GARP as a novel tumor antigen in ATL. The anti-GARP mAb DS-1055a selectively and effectively depleted malignant ATL cells, supporting the proof-of-concept in the preclinical study. Notably, GARP is crucial not only as a specific target of antibody therapy but also for essential functions such as phenotyping and survival of ATL cells via TGF-β. Thus, the GARP–TGF-β pathway is a critical axis for understanding ATL origin and the development of unprecedented bidirectional therapeutic strategies.

Previously, we have shown that HTLV-1-infected and tumor cell-specific transcriptome data are useful for understanding the pathogenesis and novel therapeutic targets in ATL [10, 32–35]. Recent molecular biological and clinical studies have demonstrated that the EZH1/2-dependent epigenetic property is a promising therapeutic target for malignant T-cell lymphomas, including ATL, supporting the idea that controlling the underlying transcriptome and biological cascades is a promising strategy against this intractable disease [15, 36]. This study focused on the characteristics and overall Treg-type gene profiles from the precancerous state through detailed analyses focused on the T-cell lineage. Remarkably, within 1 month of HTLV-1 infection, the expression levels of Treg genes, including *FOXP3* and *CTLA4*, were significantly enhanced in the de novo infected cells, particularly in the presence of TGF-β. The overexpression of *LRRC32* (GARP), which activates and releases TGF-β from the precancerous state, indicates that the Treg phenotype may be fixed by GARP–TGF-β after induced HTLV-1 infection and associated changes in viral protein expression and chromatin structure. Indeed, several studies have reported that GARP and TGF-β control Treg phenotype stability [1, 37–39].

TGF-β controls diverse cellular processes, and the detailed molecular mechanisms of the TGF-β signaling pathway have been elucidated. A recent study revealed a dynamic allosteric mechanism of autocrine TGF-β signaling without release [40]. TGF-β is generally a key factor in Treg induction and has an immunosuppressive function. However, its role is controversial and challenging to define uniformly, given its pleiotropic and context-dependent activity. So far, in the context of ATL, the mechanism of resistance to the growth-inhibitory effect of TGF-β by viral proteins Tax [41] or inhibitory Smad7 [42] and the proliferative effect via the activation of the Smad pathway by HBZ [43] have been reported. In the present study, exogenous TGF-β was found to play a direct role in ATL cell proliferation with changes in cell signaling activities and transcriptome. TGF-β also induced the activation of noncanonical pathways associated with ATL cell proliferation, indicating that the basis of ATL may account for the differential effects of TGF-β. Moreover, one of the de novo infected cells expanded clonally with continuous treatment with TGF-β. Infected cells sensitive to TGF-β might survive by competing with other infected cells, suggesting that a similar environment favorable to the survival of Treg-type ATL cells was established in asymptomatic carriers and patients with ATL. For the controversial immunosuppressive mechanism in ATL, we have provided the novel GARP–TGF-β axis as a cell contact-independent suppressive function. Further comprehensive studies on Treg-type ATL are needed to elucidate the molecular mechanisms by which GARP–TGF-β with other Treg factors, such as the cell surface molecules CTLA4 and TIGIT, may be involved in phenotyping, clonal proliferation, and tumorigenesis.

While GARP mRNA levels were similarly elevated in both premalignant infected cells and malignant ATL cells, surface GARP expression showed a gradient across disease progression, with abnormally higher levels in ATL cells than in infected cells. It should be noted that infected cells from asymptomatic carriers also showed higher surface GARP expression compared to uninfected cells. Among ATL cases, surface GARP expression varied and tended to be particularly high in clinically aggressive cases, suggesting that surface GARP may increase as ATL progresses. In general, although GARP mRNA is expressed by multiple cell types, surface GARP has been detected in a limited number of cell types [44]. Possible mechanisms underlying the gap in surface GARP expression between carriers and patients with ATL include: (1) aberrant activation of TCR signaling, which is required for efficient surface localization of GARP [45]; and (2) suppression of LAPTM4B, a known negative regulator of GARP expression [46], in ATL. Indeed, some ATL cases with extremely high surface GARP expression showed abnormal signal activation due to genetic alterations in the TCR pathway (Supplementary Fig. 2b) [7]. Furthermore, our previous data suggested that the *LAPTM4B* locus is epigenetically suppressed in ATL cells [15]. While some carriers showed a similar trend, their profiles were quantitatively distinct from those of patients with ATL. A detailed elucidation of the regulatory mechanisms governing GARP expression is a significant next issue not only in the context of ATL pathogenesis, but also in broader immunological research.

Consistent with our results, GARP is expressed in activated highly suppressive Tregs, which form the major suppressive fraction of Tregs in the tumor microenvironment, whereas GARP expression is limited in other effector T-cell lineages [47]. Several GARP-targeting antibodies are being developed with clinical promise as a Treg-specific treatment approach [44, 48]. DS-1055a, a mAb with enhanced ADCC activity against human GARP, has been developed for augmentation of antitumor immunity by depleting highly suppressive Tregs [47]. In ATL, the tumor cells show Treg-like phenotype and express GARP, which is expected to have a dual effect: a direct antitumor effect and the activation of tumor immunity. Because GARP also releases TGF-β in ATL cells, DS-1055a can suppress TGF-β secretion and indirectly activate tumor immunity. ADCC antibody therapy has already demonstrated clinical efficacy against ATL [13]. CCR4, the target of mogamulizumab, is a highly expressed competent therapeutic target but somewhat lacks the functional importance and specificity of a Treg [49, 50]. Cell surface GARP does not show a broad expression as CCR4, which is already a therapeutic target [13, 49], but is markedly upregulated in more malignant cells and is expected to have sufficient functional importance and specificity. Identifying the new targetable antigen will expand therapeutic strategies against ATL. DS-1055a is expected to efficiently combat ATL with minimal side effects by specifically and preferentially depleting the core cell population of ATL. DS-1055a not only induced robust and specific ADCC but also partially induced ADCC against GARP-negative and dim populations based on the plastic cell surface expression of GARP, suggesting that long-term administration of DS-1055a could deplete the entire ATL cell population. Given the possibility that some ATL cells may persist under GARP-targeting therapy, it will be needed to explore complementary and optimized strategies, such as combination therapies, to eradicate the entire tumor burden. GARP is an emerging target for tumor therapy; therefore, further studies on the dynamic regulation of GARP and related pathological function will provide a deeper understanding of the mechanism of ATL progression and further lead to therapeutic efficacy in targeting activated Tregs in other cancer types. The insights from this study may be the key to solving two tough questions: the origin of ATL and the challenging immunological strategies against intractable diseases.

## Supplementary information

Supplemental text

Supplemental figures

## Data Availability

All sequencing data have been deposited in the National Bioscience Database Center (NBDC) Human Database under the accession number JGAS000553 (https://humandbs.dbcls.jp/en/hum0252-v3).

## References

[1] Moreau JM, Velegraki M, Bolyard C, Rosenblum MD, Li Z. Transforming growth factor–β1 in regulatory T cell biology. Sci Immunol. 2022;7:eabi4613. 35302863 10.1126/sciimmunol.abi4613 PMC10552796

[2] Tanaka A, Sakaguchi S. Regulatory T cells in cancer immunotherapy. Cell Res. 2017;27:109–18. 27995907 10.1038/cr.2016.151 PMC5223231

[3] Chen B-J, Zhao J-W, Zhang D-H, Zheng A-H, Wu G-Q. Immunotherapy of cancer by targeting regulatory T cells. Int Immunopharmacol. 2022;104:108469. 35008005 10.1016/j.intimp.2021.108469

[4] Ohue Y, Nishikawa H. Regulatory T (Treg) cells in cancer: can Treg cells be a new therapeutic target?. Cancer Sci. 2019;110:2080–9. 31102428 10.1111/cas.14069 PMC6609813

[5] Iwanaga M, Watanabe T, Yamaguchi K. Adult T-cell leukemia: a review of epidemiological evidence. Front Microbiol. 2012;3:322. 22973265 10.3389/fmicb.2012.00322 PMC3437524

[6] Imaizumi Y, Iwanaga M, Nosaka K, Ishitsuka K, Ishizawa K, Ito S, et al. Prognosis of patients with adult T-cell leukemia/lymphoma in Japan: a nationwide hospital-based study. Cancer Sci. 2020;111:4567–80. 32976684 10.1111/cas.14658 PMC7734015

[7] Kataoka K, Nagata Y, Kitanaka A, Shiraishi Y, Shimamura T, Yasunaga J, et al. Integrated molecular analysis of adult T cell leukemia/lymphoma. Nat Genet. 2015;47:1304–15. 26437031 10.1038/ng.3415

[8] Yamagishi M, Fujikawa D, Watanabe T, Uchimaru K. HTLV-1-mediated epigenetic pathway to adult T-cell leukemia-lymphoma. Front Microbiol. 2018;9:1686. 30087673 10.3389/fmicb.2018.01686 PMC6066519

[9] Watanabe T. Adult T-cell leukemia: molecular basis for clonal expansion and transformation of HTLV-1–infected T cells. Blood. 2017;129:1071–81. 28115366 10.1182/blood-2016-09-692574 PMC5374731

[10] Mizuike J, Suzuki K, Tosaka S, Kuze Y, Kobayashi S, Nakashima M, et al. Rewired chromatin structure and epigenetic gene dysregulation during HTLV-1 infection to leukemogenesis. Cancer Sci. 2025;116:513–23. 39561277 10.1111/cas.16388 PMC11786301

[11] Ishitsuka K, Tamura K. Human T-cell leukaemia virus type I and adult T-cell leukaemia-lymphoma. Lancet Oncol. 2014;15:e517–26. 25281470 10.1016/S1470-2045(14)70202-5

[12] El Hajj H, Tsukasaki K, Cheminant M, Bazarbachi A, Watanabe T, Hermine O. Novel treatments of adult T Cell leukemia lymphoma. Front Microbiol. 2020;11:1062. 32547515 10.3389/fmicb.2020.01062 PMC7270167

[13] Ishida T, Joh T, Uike N, Yamamoto K, Utsunomiya A, Yoshida S, et al. Defucosylated anti-CCR4 monoclonal antibody (KW-0761) for relapsed adult T-Cell leukemia-lymphoma: a multicenter phase II study. J Clin Oncol. 2012;30:837–42. 22312108 10.1200/JCO.2011.37.3472

[14] Horwitz S, O’Connor OA, Pro B, Illidge T, Fanale M, Advani R, et al. Brentuximab vedotin with chemotherapy for CD30-positive peripheral T-cell lymphoma (ECHELON-2): a global, double-blind, randomised, phase 3 trial. Lancet. 2019;393:229–40. 30522922 10.1016/S0140-6736(18)32984-2 PMC6436818

[15] Yamagishi M, Kuze Y, Kobayashi S, Nakashima M, Morishima S, Kawamata T, et al. Mechanisms of action and resistance in histone methylation-targeted therapy. Nature. 2024;627:221–8. 38383791 10.1038/s41586-024-07103-x PMC10917674

[16] Karube K, Ohshima K, Tsuchiya T, Yamaguchi T, Kawano R, Suzumiya J, et al. Expression of FoxP3, a key molecule in CD4+CD25+ regulatory T cells, in adult T-cell leukaemia/lymphoma cells. Br J Haematol. 2004;126:81–4. 15198736 10.1111/j.1365-2141.2004.04999.x

[17] Toulza F, Nosaka K, Takiguchi M, Pagliuca T, Mitsuya H, Tanaka Y, et al. FoxP3+ regulatory T cells are distinct from leukemia cells in HTLV-1–associated adult T-cell leukemia. Int J Cancer. 2009;125:2375–82. 19544530 10.1002/ijc.24664

[18] Uchiyama T, Hori T, Tsudo M, Wano Y, Umadome H, Tamori S, et al. Interleukin-2 receptor (Tac antigen) expressed on adult T cell leukemia cells. J Clin Invest. 1985;76:446–53. 2993359 10.1172/JCI111992 PMC423837

[19] Satou Y, Yasunaga J, Zhao T, Yoshida M, Miyazato P, Takai K, et al. HTLV-1 bZIP factor induces T-cell lymphoma and systemic inflammation in vivo. PLoS Pathog. 2011;7:e1001274. 21347344 10.1371/journal.ppat.1001274 PMC3037353

[20] Zhao T, Satou Y, Sugata K, Miyazato P, Green PL, Imamura T, et al. HTLV-1 bZIP factor enhances TGF-β signaling through p300 coactivator. Blood. 2011;118:1865–76. 21705495 10.1182/blood-2010-12-326199 PMC3158717

[21] Koya J, Saito Y, Kameda T, Kogure Y, Yuasa M, Nagasaki J, et al. Single-cell analysis of the multicellular ecosystem in viral carcinogenesis by HTLV-1. Blood Cancer Discov. 2021;2:450–67. 34661162 10.1158/2643-3230.BCD-21-0044 PMC8514013

[22] Tan BJY, Sugata K, Reda O, Matsuo M, Uchiyama K, Miyazato P, et al. HTLV-1 infection promotes excessive T cell activation and transformation into adult T cell leukemia/lymphoma. J Clin Invest. 2021;131:e150472. 34907908 10.1172/JCI150472 PMC8670839

[23] Chen S, Ishii N, Ine S, Ikeda S, Fujimura T, Ndhlovu LC, et al. Regulatory T cell-like activity of Foxp3+ adult T cell leukemia cells. Int Immunol. 2006;18:269–77. 16361311 10.1093/intimm/dxh366

[24] Ono M, Satou Y. Spectrum of Treg and self-reactive T cells: single cell perspectives from old friend HTLV-1. Discov Immunol. 2024;3:kyae006. 38863793 10.1093/discim/kyae006 PMC11165433

[25] Shimoyama M. Diagnostic criteria and classification of clinical subtypes of adult T-cell leukaemia-lymphoma. a report from the Lymphoma Study Group (1984-87). Br J Haematol. 1991;79:428–37. 1751370 10.1111/j.1365-2141.1991.tb08051.x

[26] Yamagishi M, Kubokawa M, Kuze Y, Suzuki A, Yokomizo A, Kobayashi S, et al. Chronological genome and single-cell transcriptome integration characterizes the evolutionary process of adult T cell leukemia-lymphoma. Nat Commun. 2021;12:4821. 34376672 10.1038/s41467-021-25101-9 PMC8355240

[27] Kobayashi S, Nakano K, Watanabe E, Ishigaki T, Ohno N, Yuji K, et al. CADM1 expression and stepwise downregulation of CD7 are closely associated with clonal expansion of HTLV-I-infected cells in adult T-cell leukemia/lymphoma. Clin Cancer Res. 2014;20:2851–61. 24727323 10.1158/1078-0432.CCR-13-3169

[28] Iwanaga M, Watanabe T, Utsunomiya A, Okayama A, Uchimaru K, Koh K-R, et al. Human T-cell leukemia virus type I (HTLV-1) proviral load and disease progression in asymptomatic HTLV-1 carriers: a nationwide prospective study in Japan. Blood. 2010;116:1211–9. 20448111 10.1182/blood-2009-12-257410

[29] Nakagawa M, Shaffer AL, Ceribelli M, Zhang M, Wright G, Huang DW, et al. Targeting the HTLV-I-regulated BATF3/IRF4 transcriptional network in adult T cell leukemia/lymphoma. Cancer Cell. 2018;34:286–97.e10. 30057145 10.1016/j.ccell.2018.06.014 PMC8078141

[30] Higuchi Y, Yasunaga J, Mitagami Y, Tsukamoto H, Nakashima K, Ohshima K, et al. HTLV-1 induces T cell malignancy and inflammation by viral antisense factor-mediated modulation of the cytokine signaling. Proc Natl Acad Sci USA. 2020;117:13740–9. 32471947 10.1073/pnas.1922884117 PMC7306771

[31] Haupt S, Söntgerath VSA, Leipe J, Schulze-Koops H, Skapenko A. Methylation of an intragenic alternative promoter regulates transcription of GARP. Biochim Biophys Acta. 2016;1859:223–34. 26584734 10.1016/j.bbagrm.2015.11.003

[32] Yamagishi M, Suzuki Y, Watanabe T, Uchimaru K. Clonal selection and evolution of HTLV-1-infected cells driven by genetic and epigenetic alteration. Viruses. 2022;14:587. 35336993 10.3390/v14030587 PMC8950914

[33] Yamagishi M, Hori M, Fujikawa D, Ohsugi T, Honma D, Adachi N, et al. Targeting excessive EZH1 and EZH2 activities for abnormal histone methylation and transcription network in malignant lymphomas. Cell Rep. 2019;29:2321–37.e7. 31747604 10.1016/j.celrep.2019.10.083

[34] Yamagishi M, Nakano K, Miyake A, Yamochi T, Kagami Y, Tsutsumi A, et al. Polycomb-mediated loss of miR-31 activates NIK-dependent NF-κB pathway in adult T Cell leukemia and other cancers. Cancer Cell. 2012;21:121–35. 22264793 10.1016/j.ccr.2011.12.015

[35] Fujikawa D, Nakagawa S, Hori M, Kurokawa N, Soejima A, Nakano K, et al. Polycomb-dependent epigenetic landscape in adult T-cell leukemia. Blood. 2016;127:1790–802. 26773042 10.1182/blood-2015-08-662593

[36] Izutsu K, Makita S, Nosaka K, Yoshimitsu M, Utsunomiya A, Kusumoto S, et al. An open-label, single-arm phase 2 trial of valemetostat for relapsed or refractory adult T-cell leukemia/lymphoma. Blood. 2023;141:1159–68. 36150143 10.1182/blood.2022016862 PMC10651775

[37] Wang J, Zhao X, Wan YY. Intricacies of TGF-β signaling in Treg and Th17 cell biology. Cell Mol Immunol. 2023;20:1002–22. 37217798 10.1038/s41423-023-01036-7 PMC10468540

[38] Lehmkuhl P, Gentz M, Garcia de Otezya AC, Grimbacher B, Schulze-Koops H, Skapenko A. Dysregulated immunity in PID patients with low GARP expression on Tregs due to mutations in LRRC32. Cell Mol Immunol. 2021;18:1677–91. 34059789 10.1038/s41423-021-00701-z PMC8245512

[39] Marie JC, Letterio JJ, Gavin M, Rudensky AY. TGF-β1 maintains suppressor function and Foxp3 expression in CD4+CD25+ regulatory T cells. J Exp Med. 2005;201:1061–7. 15809351 10.1084/jem.20042276 PMC2213134

[40] Jin M, Seed RI, Cai G, Shing T, Wang L, Ito S, et al. Dynamic allostery drives autocrine and paracrine TGF-β signaling. Cell. 2024;187:6200–19.e23. 39288764 10.1016/j.cell.2024.08.036 PMC11531391

[41] Mori N, Morishita M, Tsukazaki T, Giam C-Z, Kumatori A, Tanaka Y, et al. Human T-cell leukemia virus type I oncoprotein Tax represses Smad-dependent transforming growth factor β signaling through interaction with CREB-binding protein/p300. Blood. 2001;97:2137–44. 11264182 10.1182/blood.v97.7.2137

[42] Nakahata S, Yamazaki S, Nakauchi H, Morishita K. Downregulation of ZEB1 and overexpression of Smad7 contribute to resistance to TGF-β1-mediated growth suppression in adult T-cell leukemia/lymphoma. Oncogene. 2010;29:4157–69. 20514018 10.1038/onc.2010.172

[43] Shichijo T, Yasunaga J, Sato K, Nosaka K, Toyoda K, Watanabe M, et al. Vulnerability to APOBEC3G linked to the pathogenicity of deltaretroviruses. Proc Natl Acad Sci USA. 2024;121:e2309925121. 38502701 10.1073/pnas.2309925121 PMC10990082

[44] Zimmer N, Trzeciak ER, Graefen B, Satoh K, Tuettenberg A. GARP as a therapeutic target for the modulation of regulatory T cells in cancer and autoimmunity. Front Immunol. 2022;13:928450. 35898500 10.3389/fimmu.2022.928450 PMC9309211

[45] Zhou AX, Kozhaya L, Fujii H, Unutmaz D. GARP–TGF-β complexes negatively regulate regulatory T cell development and maintenance of peripheral CD4+ T cells in vivo. J Immunol. 2013;190:5057–64. 23576681 10.4049/jimmunol.1300065 PMC3653571

[46] Huygens C, Liénart S, Dedobbeleer O, Stockis J, Gauthy E, Coulie PG, et al. Lysosomal-associated transmembrane protein 4B (LAPTM4B) decreases transforming growth factor β1 (TGF-β1) production in human regulatory T cells. J Biol Chem. 2015;290:20105–16. 26126825 10.1074/jbc.M115.655340 PMC4536422

[47] Satoh K, Kobayashi Y, Fujimaki K, Hayashi S, Ishida S, Sugiyama D, et al. Novel anti-GARP antibody DS-1055a augments anti-tumor immunity by depleting highly suppressive GARP+ regulatory T cells. Int Immunol. 2021;33:435–46. 34235533 10.1093/intimm/dxab027

[48] Cuende J, Liénart S, Dedobbeleer O, van der Woning B, De Boeck G, Stockis J, et al. Monoclonal antibodies against GARP/TGF-β1 complexes inhibit the immunosuppressive activity of human regulatory T cells in vivo. Sci Transl Med. 2015;7:284ra56. 25904740 10.1126/scitranslmed.aaa1983

[49] Ishii T, Ishida T, Utsunomiya A, Inagaki A, Yano H, Komatsu H, et al. Defucosylated humanized anti-CCR4 monoclonal antibody KW-0761 as a novel immunotherapeutic agent for adult T-cell leukemia/lymphoma. Clin Cancer Res. 2010;16:1520–31. 20160057 10.1158/1078-0432.CCR-09-2697

[50] Maeda Y, Wada H, Sugiyama D, Saito T, Irie T, Itahashi K, et al. Depletion of central memory CD8+ T cells might impede the antitumor therapeutic effect of Mogamulizumab. Nat Commun. 2021;12:7280. 34907192 10.1038/s41467-021-27574-0 PMC8671535

