# Supplementary material for: Bidirectional anti-tumor and immunological strategies by targeting GARP–TGF-β axis in adult T-cell leukemia/lymphoma
Source: Leukemia. 2025 Aug 4;39(10):2465–76. doi: 10.1038/s41375-025-02725-0 (PMC12463658; doi:10.1038/s41375-025-02725-0)
Supplement: Supplementary file 1 — Supplemental text [file 41375_2025_2725_MOESM1_ESM.pdf]

Supplementary Tables

Supplementary Table 1: Oligo DNA and PCR primers used in the study.

Insert Sequences for Lentiviral Vector (Name: Sequence) (Underlines represent antisense of target sequences)

Name	Sequence
LV-shGARP#1-top	5'-GAT CCC CAT CTG AGC TTG TCT AAC AAA CGT GTG CTG TCC GTT TGT TGG ACA AGT TCA GGT TTT TTG GAA AT-3'
LV-shGARP#1-bottom	5'-CTA GAT TTC CAA AAA ACC TGA ACT TGT CCA ACA AAC GGA CAG CAC ACG TTT GTT AGA CAA GCT CAG ATG GG-3'
LV-shGARP#2-top	5'-GAT CCC CGA GTC TGG ATT TGA GTT ACA AAC GTG TGC TGT CCG TTT GTA GCT CAA ATC CAG ATT CTT TTT GGA AAT-3'
LV-shGARP#2-bottom	5'-CTA GAT TTC CAA AAA GAA TCT GGA TTT GAG CTA CAA ACG GAC AGC ACA CGT TTG TAA CTC AAA TCC AGA CTC GGG-3'
LV-shGARP#3-top	5'-GAT CCC CGT AGA AGT TTG ACC AGC AGT AAC GTG TGC TGT CCG TTA CTG TTG GTT AAA CTT CTG CTT TTT GGA AAT-3'
LV-shGARP#3-bottom	5'-CTA GAT TTC CAA AAA GCA GAA GTT TAA CCA ACA GTA ACG GAC AGC ACA CGT TAC TGC TGG TCA AAC TTC TAC GGG-3'
LV-shGARP#4-top	5'-GAT CCC CGT AGG CTG TTT GTC GCG TCT TAC GTG TGC TGT CCG TAA GAC GTG ACA AAC AGT CTG CTT TTT GGA AAT-3'
LV-shGARP#4-bottom	5'-CTA GAT TTC CAA AAA GCA GAC TGT TTG TCA CGT CTT ACG GAC AGC ACA CGT AAG ACG CGA CAA ACA GCC TAC GGG-3'

Quantitative PCR Primer List (Target Gene: Sequence)

Gene symbol	Forward	Reverse
LRRC32 (GARP)	5'-GGGAACATTTGCTTTGGAGA-3'	5'-AGCAGGATCTGGGGTCTCA-3'

Supplementary Figures

Supplementary Fig. 1: Abnormal Treg-type gene expression profiles of each case.

(a) Heatmaps and hierarchical clustering of expression patterns of Treg-downregulated genes in uninfected cells (P, $CD4^{+}/CADM^{-}/CD7^{+}$), infected cells (D, $CD4^{+}/CADM1^{+}/CD7^{+}$), and ATL cells (N, $CD4^{+}/CADM1^{+}/CD7^{-}$) from asymptomatic carriers (AC, n = 13) and ATL cells from patients with indolent type ATL (iATL, n = 9) and acute type ATL (aATL, n = 13). Genes with significant differences between aATL_N and normal $CD4^{+}$ T cells are shown in red (upregulated) or blue (downregulated) ($P < 0.05$). (b) PCA clustering of Treg gene expression in AC_P (n = 5), AC_D (n = 8), iATL_N (n = 9), aATL_N (n = 13) and normal $CD4^{+}$ T-cells (n = 3). (c) Heat map and hierarchical clustering of expression patterns of Treg-associated genes in each individual. (d) FOXP3, TIGIT, and PD-1 staining of PBMC from ATL #2 and ATL #7.

Supplementary Fig. 2: Detailed information of primary ATL cells.

(a) Bar graph shows relative promoter sum at the *LRRC32* locus in three ATL cases. (b) Pie charts represent clonalities of ATL cells estimated by high-throughput sequencing based mapping of proviral integration sites. Genes detected somatic mutations in ATL cells.

Supplementary Fig. 3: GARP-TGF-β pathway promotes the proliferation of ATL cells.

(a) Knockdown of GARP in C91/PL cells. Bar graphs show knockdown efficiencies of GARP by flow-cytometry and qRT-PCR. Line graph shows percentage of each Venus-positive population transduced with shRNA series. (b) Bar graph shows the proliferative effects of GARP knockdown and ectopic expression in C91/PL cells. (c) Simultaneous knockdown of GARP with shGARP#4, which targets the 3’ UTR, and ectopic expression of the GARP coding sequence via lentiviral vector in C91/PL cells. Cell surface GARP expression was evaluated by flow cytometry. (d) Overexpression of GARP in C91/PL cells. Cell surface GARP expression was evaluated by flow cytometry. (e) Pie charts represent clonalities of PBMCs from patients with ATL (n = 3) under three conditions: primary, cultured without TGF-β, and cultured with TGF-β, estimated by high-throughput sequencing based mapping of proviral integration sites. Line graph shows the percentage of the major clones (top two clones combined). (f) Histogram shows TGF-β receptor II (TGF-βRII) expression in PBMC from ATL #3 gated on “P” and “N” subpopulations. Box plot shows TGF-βRII MFI in PBMC from ATL patients (n = 3) gated on “P” and “N” subpopulations. (g) Raw data for Figure 3G. Dotted areas denote blots’ image included in the manuscript.

Supplementary Fig. 4: TGF-β suppresses the activities of effector T-cells in a concentration-dependent manner.

(a) Ki-67 and IFN-γ staining gated on $CD4^+$ or $CD8^+$ subpopulations. PBMCs were treated with recombinant human TGF-β at each concentration. Line graphs show Ki-67 MFI (left) and IFN-γ positive cells (%) (right) in $CD4^+$ or $CD8^+$ T-cells.

Supplementary Fig. 5: The plasticity of surface GARP expression.

(a) ADCC assays were performed on PBMCs from patients with ATL (n = 5) using DS-1055a in the presence of effector NK cells from healthy donors for 4 and 24 hours. ADCC activities were evaluated by detection of the dead ATL cells ($CD4^+$/$CADM1^+$/7-AAD^+). (b) Line graph shows the relative proportion of dead ATL cells induced by DS-1055a on days 3 and 7 in ATL case #5 used in Fig. 5b and Supplementary Fig. 5a. (c) ADCC assays were performed on an engineered venus-labeled GARP $^{dim+}$ C91/PL cells using DS-1055a in the presence of effector PBMCs from healthy donors. ADCC activities were evaluated by detection of $venus^+$ cell population using flow cytometry. (d) Line graph shows the percentage of $GARP^+$ cells after sorting of $GARP^+$ or $GARP^-$ C91/PL cells. They were cultured for one month. The expression level of GARP was measured by flow cytometer.
