# Supplementary material for: Bidirectional anti-tumor and immunological strategies by targeting GARP–TGF-β axis in adult T-cell leukemia/lymphoma
Source: Leukemia. 2025 Aug 4;39(10):2465–76. doi: 10.1038/s41375-025-02725-0 (PMC12463658; doi:10.1038/s41375-025-02725-0)

Supplementary Fig. 1

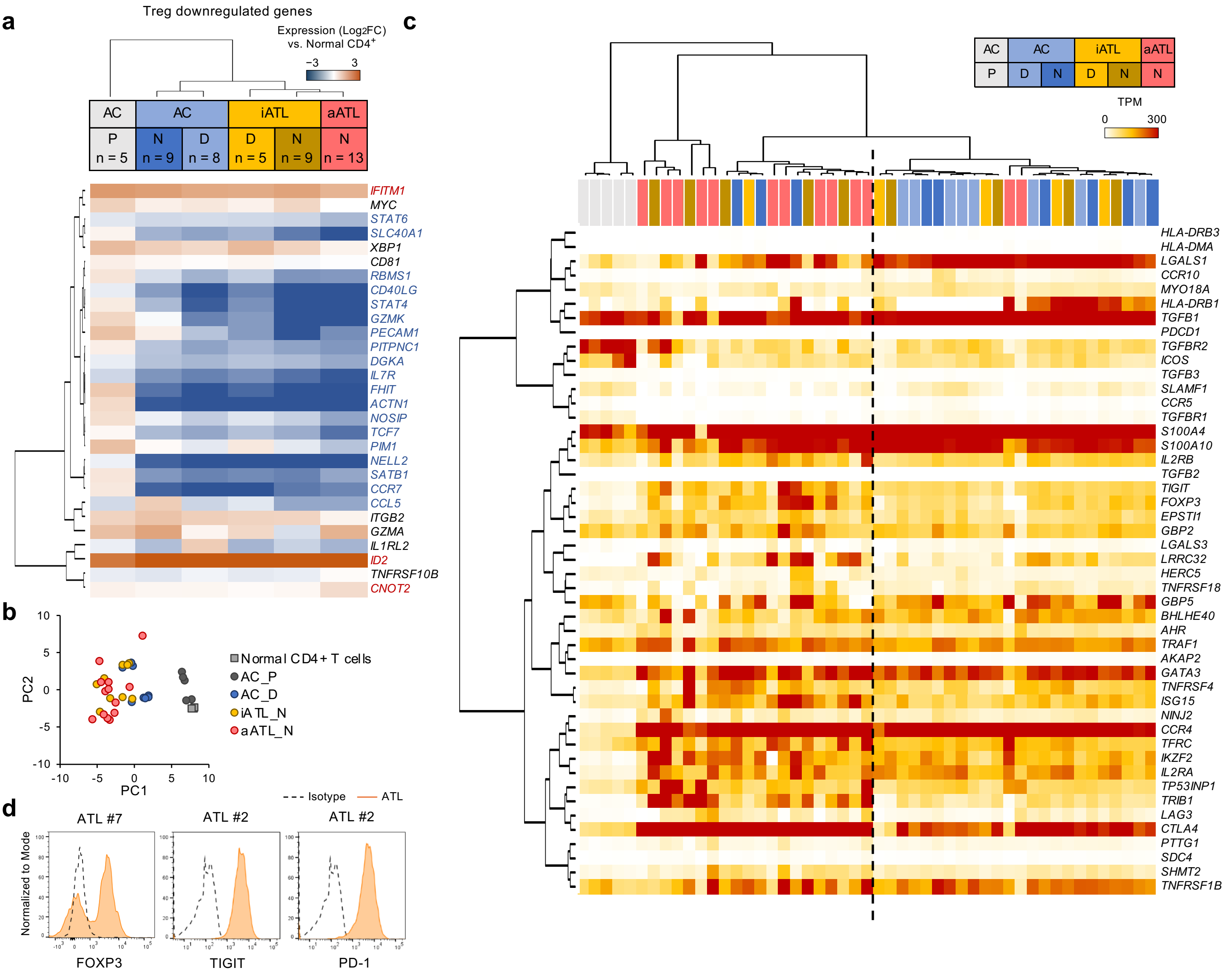

Supplementary Fig. 2

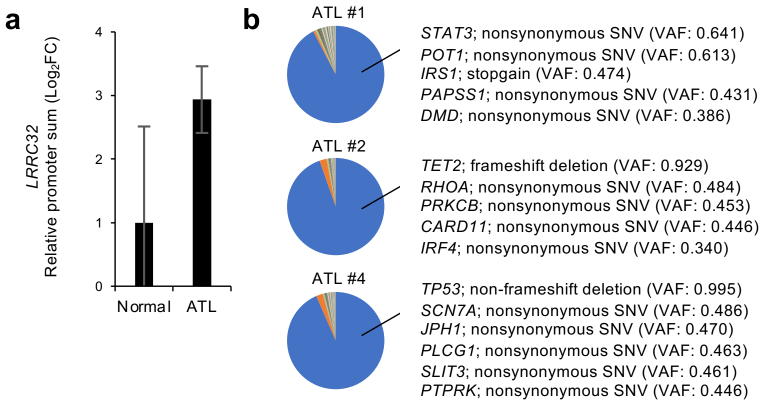

Supplementary Fig. 3

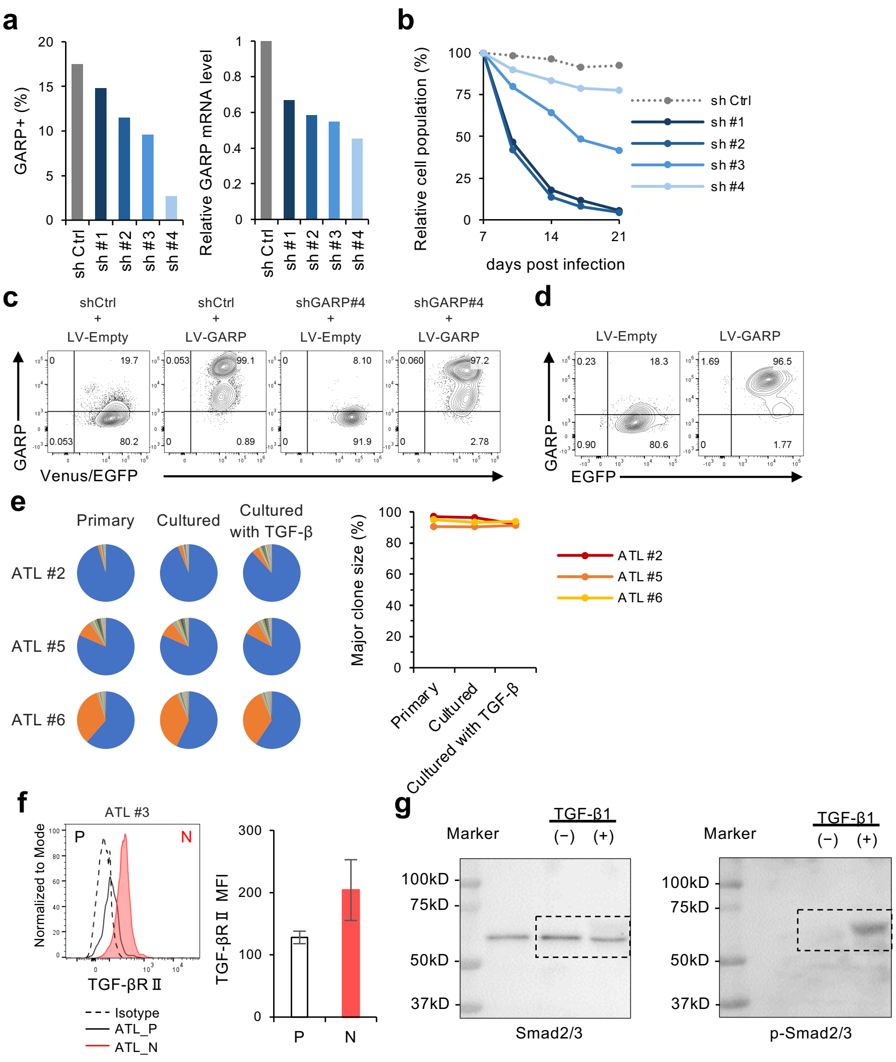

Supplementary Fig. 4

a

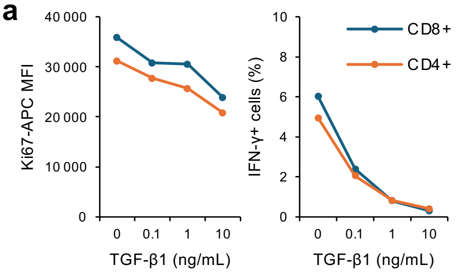

Supplementary Fig. 5

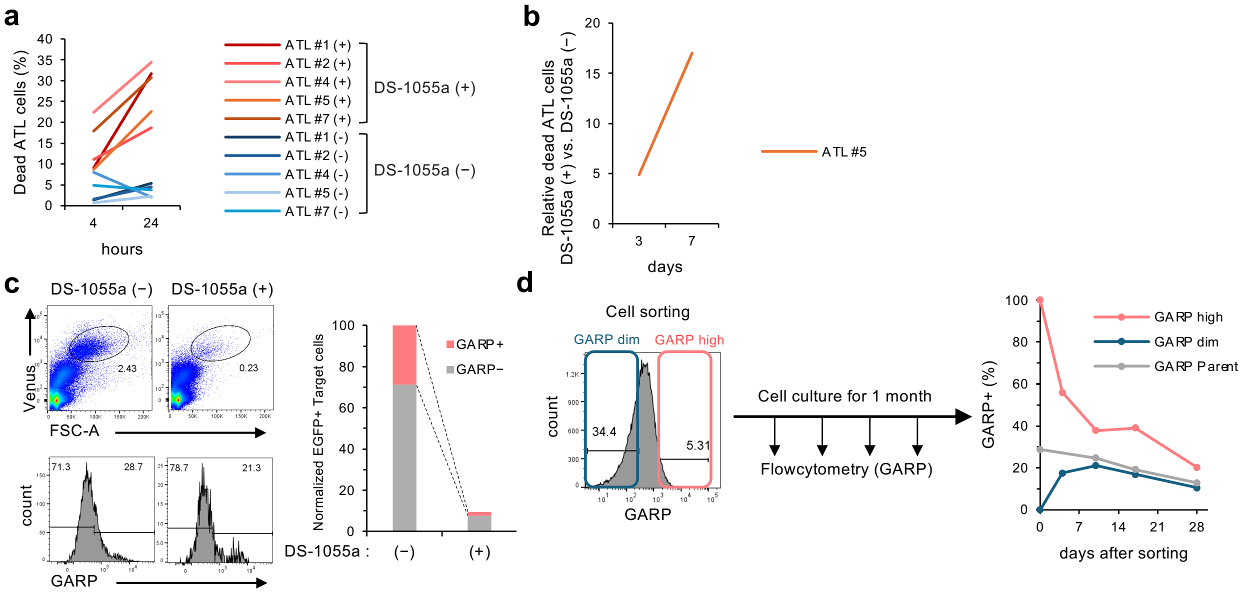

Supplement: Supplementary file 2 — Supplemental figures [file 41375_2025_2725_MOESM2_ESM.pdf]
